# The Uncharted Territories of Esophageal Cancer with Cardiac and Skeletal Muscle Metastasis: A Case Report and Literature Review

**DOI:** 10.3390/medicina60071146

**Published:** 2024-07-16

**Authors:** Hala Hassanain, Omar Hassanain, Maen Abdelrahim

**Affiliations:** 1 Department of Internal Medicine, Houston Methodist Hospital, Houston, TX 77030, USA; 2 Burroughs Wellcome Fund Fellow, Texas A&M Academy of Physician Scientists, Houston TX 77030, USA; 3 Department of General Surgery, Swedish Medical Center, Englewood, CO 80113, USA; 4 Section of GI Oncology, Department of Medical Oncology, Houston Methodist Neal Cancer Center, Houston, TX 77429, USA

**Keywords:** esophageal cancer, immunotherapy, distant metastasis, check point inhibitors, ctDNA

## Abstract

*Background:* Esophageal cancer (EC) comprises 1% of all diagnosed cancers in the USA. It is more common in other parts of the world. If there is distant metastasis, the relative survival rate is 6%. There are no standardized screening methods for EC. *Case Presentation:* We reported a four-year case of esophageal cancer, a P53-positive mutation with atypical distant metastasis to the cardiac and skeletal muscles. The patient was managed with multimodal therapy, including immunotherapy, which could have been a factor in prolonged survival. *Conclusions:* Distant metastases are typically seen postmortem, and with prolonged survival, we are able to find such unique metastases antemortem. Despite a history of negative scans, the patient’s ctDNA (circulating tumor DNA) remained positive, which was a better predictor of recurrence in this case. Future research is required to establish cost-effective screening methods and standardized treatments.

## 1. Introduction

Esophageal cancer (EC) is the sixth most common cause of cancer death worldwide [1]. It is more prevalent among males than females and makes up about 1% of all cancers in the USA. The lifetime risk of EC in the USA is 1:125 in men and about 1:417 in women [2]. It is more common in White people, and the associated subtype is adenocarcinoma. However, the most common histological subtype worldwide is squamous cell carcinoma (SCC). EC is an aggressive cancer usually diagnosed at advanced stages at presentations [3]. To this date, we do not have established screening guidelines for EC. Moreover, noninvasive diagnostic and surveillance tools such as ctDNA, which could provide a snapshot of tumor activity before solid tumor formation, are not well studied for EC. The common sites of metastasis of EC include lymph nodes, lungs, liver, adrenals, bones, and brain. Unexpected metastasis sites are rare in EC due to its unique anatomy and complex plexuses. Chemo-immunotherapy remains the standard of care for advanced stages of EC.

## 2. Case Presentation

**Figure 1:**
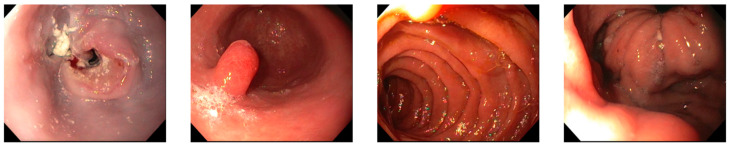
EGD showing a partially obstructing, ulcerating mass in the middle 1/3rd of the esophagus, and a medium-sized hiatal hernia.

A 70-year-old female with a past medical history of obesity s/p gastric banding presented to the clinic in 2019 due to a one-month history of nausea and vomiting followed by difficulty swallowing and feeling as though her esophagus was “swollen” General surgery evaluated the patient to have her gastric bands removed. An EDG (Esophagogastroduodenoscopy) was performed, which was unremarkable. The barium swallow study was negative, and she denied the proposed gastric bypass surgery at the time and was lost to follow-up due to family stressors. She was seen by ENT and treated for chronic reflux without any relief. She presented it again to outpatient Internal Medicine a year later with worsening mechanical dysphagia and belching. Her physical exam was unremarkable, and she was referred to EGD and colonoscopy for iron deficiency anemia, which showed a partially obstructing, ulcerating mass in the middle 1/3rd of the esophagus (Figure 1). Colonoscopy results were pertinent for hemorrhoids on the perianal exam, nonbleeding internal and external hemorrhoids, and sigmoid diverticulosis.

The biopsy results from the EGD confirmed invasive moderate differentiated squamous cell carcinoma (SCC) with necrosis and fungal filamentous hyphae. An initial PET/CT showed an esophageal mass with no evidence of metastatic disease. The clinical stage was unknown, with no EGD/EUS TXNO. She had locally advanced disease and underwent chemoradiation with Taxol/Carboplatin. A J tube was placed due to worsening dysphagia secondary to radiation esophagitis, which was complicated by multiple hospitalizations due to leaks. Repeat EUS/EGD conducted a year later showed a benign-appearing esophageal stenosis (likely due to radiation) at the location of the previous cancer. The PET scan also showed a marked increase in esophageal mass.

**Figure 2:**
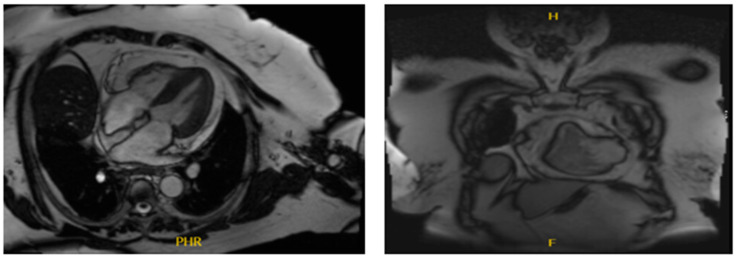
CV MRI showing a large intracardial mass (27 mm × 17 mm) in the mid-lateral LV wall with tissue characteristics suggestive of metastasis with normal biventricular size and systolic function.

**Figure 3:**
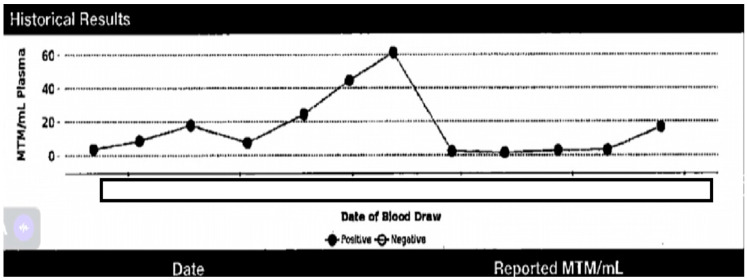
Patient’s CtDNA trend.

NGS was positive for a P53 mutation (negative for other actionable mutations such as KRAS, BRAF, NRAS, and MSI). Repeat EGD EUS performed the following year showed a single 12 mm sessile polyp with no bleeding in the gastric antrum and was biopsied (pathology showed hyperplastic polyp), candida esophagitis, and the benign-appearing esophageal stenosis. The patient had regular EGD/EUS, which was unremarkable. PET scans then showed a new mild uptake at the posterolateral aspect of the left ventricle that corresponds to a subtle, new soft tissue density on CT. However, though an unusual location, metastatic disease was suspected. No evidence of esophageal malignancy was seen then. TTE then showed no cardiac masses or any changes from the previous echo. However, the chest CT showed a mass. A cardiac MRI also confirmed a sizeable intracardial mass (27 mm × 17 mm) in the mid-lateral LV wall with tissue characteristics suggestive of metastasis with normal biventricular size and systolic function (Figure 2). She was started on FOLFOX/Pembrolizumab (mFOLFOX6 [Fluorouracil 400 mg/m^2^, fluorouracil 2400 mg/m^2^ CIV, leucovorin 400 mg/m^2^, oxaliplatin 85 mg/m^2^, infused every 14 days] with Pembrolizumab [400 mg Q6wks]) as she was referred to radiology to evaluate for SBRT to mass, which was deferred due to prior radiation-induced pneumonitis. The patient was then switched to 5FU/leucovorin/Pembrolizumab 3 months later, and a PET scan also showed mild uptake in the lower esophagus—which may be inflammatory or neoplastic, with no evidence of active metastatic disease—as well as reactive uptake in the bone marrow and spleen. CEA was trending down. CtDNA trends were obtained (Figure 3).

**Figure 4:**
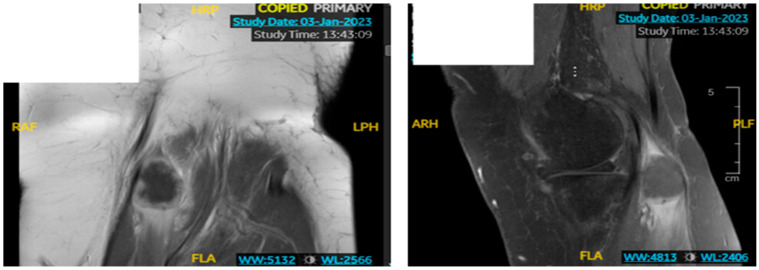
Left lower extremity MRI (Magnetic Resonance Imaging) showed a 1.8 × 1.9 × 2.6 cm circumscribed mass in the medial head of the gastrocnemius muscle at the level of the tibial plateau.

Two years from the initial diagnosis, the patient had bilateral calf pain with no other symptoms. PET/CT showed no evidence of disease. She continued to have bilateral calf pain associated with left knee pain when walking. It was associated with intermittent numbness and tingling in the LLE. She was started on gabapentin. Left lower extremity Magnetic Resonance Imaging (MRI) without contrast showed a circumscribed mass of 1.8 × 1.9 × 2.6 cm in the medial head of the gastrocnemius muscle at the level of the tibial plateau. Repeat MRI LE with contrast showed no significant change in the size of the 2 × 2.5 cm cyst nodule within the medial head of the gastrocnemius (Figure 4). There was a peripheral perilesional enhancement, confirming it was a solid tumor.

A biopsy of the left calf mass showed invasive squamous cell carcinoma, which was moderately differentiated, extensive tumor necrosis. A repeat PET/CT was conducted, and it showed a markedly avid left posterior upper thigh/knee joint mass, suspicious of malignancy. Otherwise, stable PET/CT showed no evidence of disease. She was referred to radiation oncology and received XRT for calf metastasis and completed the doses without complications. At that point, the patient was s/p 15 cycles of 5FU/Pembrolizumab and was only on Pembrolizumab, which then was switched back to 5FU/Pembrolizumab given the new calf metastasis. The most recent PET/CT showed stable findings. However, a mild uptake was seen in the gastrohepatic lymph node. An EGD/EUS showed a recurrence of the previously treated esophageal stricture. Dilation was performed, and FNA of the tissue density around the Lap-Band. The cytology was positive for metastatic esophageal cancer, and the patient received a complete course of XRT. A repeat showed concern for recurrent disease in the left gastrocnemius muscle and treated lesion inferior to the gastric banding vice with no residual disease. She was last seen in the Oncology clinic two months ago and is to follow up with PET CT to discuss the next steps in her treatment.

## 3. Discussion

The most common sites of EC metastasis are the lymph nodes, lungs, liver, bones, adrenals, and brain. In a pivotal systematic review, Shaheen, Ghibur, and Alsaid identified 10,049 articles on EC metastasis and the associated sites. In their study, they included unconventional sites of metastasis. On average, 84% of cases were men with a median age of 60.7 years, 40% were adenocarcinoma, 60% were SCC, and the OS was 10.2 months. They divided the Unexpected Metastasis (UM) sites into five anatomical places; skeletal muscle was one and the least common site of metastasis, constituting roughly 7% of all metastases (UM). Almost 50% of cases were stage IV upon initial diagnosis, and about 2/3rd originated from the lower esophagus [4].

Skeletal Muscle Metastasis (SMM) is usually a collateral of widespread disease rather than a common site of primary metastasis [5]. A few case series/reports describing SMM as a site of metastasis were identified in the literature. However, no case was ever reported of SMM being the primary site of metastasis for EC [6,7]. However, in a recent case series of a single-center experience, 205 patients with EC treated between 2006 and 2010 were studied for SMM. Four had SMM from esophageal carcinoma and two patients had SCC. One patient had SCC of the esophagus and concurrent pancreatic adenocarcinoma. The SMM was identified to be from the pancreas. In all these patients, SMM was the first manifestation of systematic disease, and the case series concluded by suggesting that the first manifestation of metastatic disease could be SMM [8].

Metastasis to the heart is rare, let alone the metastasis of tumors arising from the esophagus, particularly SCC [5,9]. The most common cardiac tumor metastases are from pleural mesothelioma, melanoma, lung adenocarcinoma, breast carcinoma, ovarian carcinoma, renal carcinoma, and pancreatic carcinoma. They are commonly found on the right side > left side of the heart and are usually small and multiple. The direct invasion of the pericardium or regional lymphatics is more common than distant metastasis. Other methods could be the bloodstream, lymphatics, and intracavitary diffusions. According to Console et al., the place where the tumor lands is crucial to the route it uses to metastasize. They continued to elaborate that if a tumor was found in the myocardium, it probably resulted from a lymphatic spread. If a tumor is in the endocardial, then it is likely from the bloodstream. EC is not one of the ones to be seen metastasizing to the heart. Overall, the unconventional distal metastasis of EC could be attributed to the abundant submucosal plexus [4]. Interestingly, most cardiac metastases are identified postmortem [5,10]. The epidemiological statistics of cardiac metastasis we have are primarily based on autopsies. If cardiac metastasis were to be symptomatic, which happens in <10% of cases, patients would develop congestive heart failure, valvular disease, arrhythmia, and, less likely, effusion [5]. According to Signorelli et al., a localized and prolonged ST-segment elevation without Q waves appears pathognomonic for myocardial tumor invasion. Cates et al. reported that the most frequently observed ECG abnormalities associated with cardiac metastasis were ST-T changes suggestive of myocardial ischemia or injury, atrial arrhythmias, and low voltage. An ECG pattern of myocardial ischemia or injury three months before death was found to be specific (96%) when looking at autopsies of patients with proven cardiac metastases [11,12]. MRI, PET, and echo remain better and more precise tools for diagnosis. 

The treatment revolution increased life expectancy. In 1960–1970, about 5% of patients survived at least five years after diagnosis. We now have a four-fold increase in survival [1]. These numbers include patients with all stages of EC. Squamous cell carcinoma (SCC) has a worse prognosis than adenocarcinoma. Biopsy remains the gold standard of diagnosis [13]. In a study that compared patients with T1 SCC vs. T1 adenocarcinoma (AC), the 5-year survival rate of patients with complete tumor removal was superior for those with adenocarcinoma (82.5%) compared with those with SCC (59.2%) (*p* < 0.03) [14]. The poorer prognosis was attributed to a higher recurrence rate and the more frequent development of second primary tumors (21% vs. 0%). Another study comparing T1 SCC and T1 AC stated that the worse prognosis is not secondary to early lymph node metastasis as postulated but is due to a four-fold higher post-op mortality and other causes attributed to surgery [15]. This, of course, is only one factor in a sea of many different aspects and could be controversial as we have advanced toward robotic surgery. Despite the advancements, we are yet to have standardized screening tests for EC [13].

Unlike HCC, pancreas, or colorectal cancers, we have no reliable noninvasive biomarker used in EC [16,17]. In a recent systematic review and meta-analysis, Chidambaram and Markar summarize the latest evidence for the clinical application of ctDNA in EC [18]. They include 15 studies that qualitatively used ctDNA and eight studies using it as a quantitative measure. The pooled sensitivity and specificity for diagnostic studies were 71.0% and 98.6%, while the pooled sensitivity and specificity for surveillance purposes were 48.9% and 95.5. Of course, ctDNA has challenges, including its short half-life of 16 min, the stabilization of the structure before testing, and its cost. No study thus far has been conducted to assess the cost-effectiveness of EC. Nonetheless, ctDNA was reported to be most helpful in lung cancer [17,18]. In our patient, despite having negative EGD/EUS for a good period of time, ctDNA remained positive and elevated with variable degrees throughout the course of treatment.

Metastatic SCC of EC is associated with a poor prognosis with a median OS of 12 months. Chemotherapy remains the standard of care in all advanced diseases regardless of histological type [19,20]. 5-fluorouracil plus platinum agent remains first-line therapy with 30–50% response rates and progression-free survival (PFS) of 4–6 months [21]. Chemotherapy is not known to provide any increased benefit in survival time but may improve QOL in selected patients [10,22,23]. In a Phase II randomized controlled trial, researchers investigated whether continuing chemotherapy would benefit patients with metastatic esophageal cancer who were not experiencing disease progression. The study concluded that while overall survival rates were similar between those who continued chemotherapy and those who stopped, there was some improvement in symptom management, such as reduced dysphagia, for those who continued the treatment [22]. In the USA, the five-year relative survival for advanced distal metastasis tumors is 4% [4].

In recent years, immunotherapies such as Pembrolizumab or Nivolumab have been widely used in many solid or liquid cancers alone or combined with chemotherapy as salvage or even as first-line therapy. In Japan, Pembrolizumab plus doublet chemotherapy is now an established first-line treatment for advanced EC after a pioneering trial called KEYNOTE-590, which was a randomized, double-blind, placebo-controlled phase III study across 168 medical centers in 26 countries. One arm of patients received intravenous Pembrolizumab (200 mg) and the other placebo, plus 5-fluorouracil and cisplatin (chemotherapy), once every three weeks for up to 35 cycles in both arms. The study concluded that the Pembrolizumab and chemotherapy arm improved the OS and PFS in patients with an EC SCC type and PD-L1 combined positive score of 10 or more, an improved OS for a previously untreated advanced SCC subtype, and an improved OS and PFS in all randomized patients regardless of histology [24].

The FDA approved Nivolumab/Chemo and Nivolumab/Ipilimumab for the Unresectable Advanced EC SCC type regardless of PD-L1 expression on 27 May 2022 [25]. The approval was based on results from the phase III CheckMate 648 trial (NCT03143153), which was assessed using Nivolumab in combination with chemotherapy or Ipilimumab vs. chemotherapy alone to treat adults with previously untreated, unrespectable advanced or metastatic esophageal squamous cell carcinoma [26].

## 4. Conclusions

Our case report demonstrated two sites of metastasis, which are generally rare for all neoplasms, particularly in esophageal cancer and more so in the SCC histological subtype. The heart and skeletal muscles are a causality of widespread metastasis rather than the primary homes of metastasis. We are noticing a slight surge in cases identifying cardiac metastasis antemortem. Despite our patient having “poor prognostic” factors, including P53, distant metastasis, and a theoretical median time to death of 5.3 months post-cardiac metastasis, she survived to live through a secondary metastasis to the skeletal muscles [5]. This leads us to believe that advancements in therapy, particularly immunotherapy and routine screening with PET and potentially ctDNA, have increased life expectancy and allowed us to shine a light on tumor metastasis sites never seen before. 

## 5. Patient Perspective

When this case report was written, the patient had received chemotherapy, radiation to the distant metastasis, and immunotherapy. She had tolerated the treatments as mentioned above with expected side effects, including nausea, vomiting, and peripheral neuropathy. Nausea did affect the quality of life for the patient, but overall, she felt that it was the right decision and continued to have complete family support. 

## Data Availability

The data of this study that supports our results are available on request from the corresponding author.
